# Quetiapine Ameliorates MIA-Induced Impairment of Sensorimotor Gating: Focus on Neuron-Microglia Communication and the Inflammatory Response in the Frontal Cortex of Adult Offspring of Wistar Rats

**DOI:** 10.3390/cells11182788

**Published:** 2022-09-07

**Authors:** Katarzyna Chamera, Katarzyna Curzytek, Kinga Kamińska, Ewa Trojan, Agnieszka Basta-Kaim

**Affiliations:** Department of Experimental Neuroendocrinology, Maj Institute of Pharmacology, Polish Academy of Sciences, 31-343 Kraków, Poland

**Keywords:** schizophrenia, maternal immune activation, sensorimotor gating, neuron–microglia axes, chlorpromazine, quetiapine, aripiprazole

## Abstract

The maternal immune activation produced by the systemic administration of lipopolysaccharide (LPS) in rats provides valuable insights into the basis of behavioural schizophrenia-like disturbances and biochemical changes in the brains of the offspring, such as microglial activation. Regarding therapy, antipsychotics continually constitute the cornerstone of schizophrenia treatment. To their various efficacy and side effects, as well as not fully recognised mechanisms of action, further characteristics have been suggested, including an anti-inflammatory action via the impact on neuron–microglia axes responsible for inhibition of microglial activation. Therefore, in the present study, we sought to determine whether chronic treatment with chlorpromazine, quetiapine or aripiprazole could influence schizophrenia-like behavioural disturbances at the level of sensorimotor gating in male offspring prenatally exposed to LPS. Simultaneously, we wanted to explore if the chosen antipsychotics display a positive impact on the neuroimmunological parameters in the brains of these adult animals with a special focus on the ligand-receptor axes controlling neuron–microglia communication as well as pro- and anti-inflammatory factors related to the microglial activity. The results of our research revealed the beneficial effect of quetiapine on deficits in sensorimotor gating observed in prenatally LPS-exposed offspring. In terms of axes controlling neuron–microglia communication and markers of microglial reactivity, we observed a subtle impact of quetiapine on hippocampal *Cx3cl1* and *Cx3cr1* levels, as well as cortical *Cd68* expression. Hence, further research is required to fully define and explain the involvement of quetiapine and other antipsychotics in *Cx3cl1*-*Cx3cr1* and/or *Cd200*-*Cd200r* axes modulation and inflammatory processes in the LPS-based model of schizophrenia-like disturbances.

## 1. Introduction

Schizophrenia is a psychiatric disorder marked by a variety of disturbances that can be typically categorised into positive and negative symptoms along with cognitive impairments [1]. The condition appears during late adolescence or early adulthood and has been associated with heredity and environmental or immunological factors, among others [2,3].

The role of inflammation in the pathophysiology of schizophrenia is well-supported by findings of altered immune parameters in both the postmortem brains [4,5,6] and the blood of patients [7,8]. Additionally, animal models of this condition employing exposure to viral or bacterial immunostimulants provide valuable insights into the basis of schizophrenia-like disturbances [9,10,11]. One of the widely implemented approaches is maternal immune activation (MIA), produced by the prenatal administration of lipopolysaccharide (LPS) [12,13,14,15,16]. When considering a neurodevelopmental model of schizophrenia, MIA with LPS has been described in terms of various behavioural disturbances, including affected sensorimotor gating [12,17,18], anxiety-like behaviour [13,19], social interactions [15], exploratory or locomotor activity [19,20,21] and cognitive deficits [22,23] as well as diverse biochemical alterations in the brains of the offspring, including the CX3CL1-CX3CR1 and CD200-CD200R pairs, which are crucial in neuron–microglia communication [13,19]. CX3CL1 is a chemokine that differs notably from other representatives of this group in both structure and role [24], whereas CD200 belongs to a class of surface antigens with immunosuppressive properties [25]. These ligands are produced mainly by neurons and bind to their corresponding receptors (CX3CR1 and CD200R, respectively) expressed by microglia [11]. Both dyads are neuroinflammatory “off” signals for microglia, whereas their dysfunctions exaggerate the proinflammatory response in the brain [26,27,28].

Microglia are major resident immune cells in the central nervous system (CNS), constituting approximately 10–15% of the total number of CNS cells [29,30]. Under physiological conditions, microglia remain in a surveillant state with the homeostatic expression of various markers as well as cytokines and chemokines [31,32]. Some pathophysiological circumstances, however, result in microglial activation, which is mediated by a wide array of cellular mechanisms [33]. Dynamic changes in microglial activity have also been reported in the course of schizophrenia, although their etiopathogenesis has not yet been conclusively explained [34,35,36].

Antipsychotics represent the cornerstone of schizophrenia therapy, leading to an overall improvement in the long-term outcomes of patients and reducing the severity and frequency of positive, negative or cognitive symptoms [37,38,39]. Generally, these drugs are grouped into two main categories, including typical (first-generation) and atypical, in which two subgroups are currently distinguished: second and third generation [40,41].

One of the most important representatives of typical antipsychotics is chlorpromazine [42]. This drug blocks dopamine (DA) D_2_ postsynaptic receptors, but it also affects serotonin (5-HT), muscarinic, α_1_-adrenergic and H_1_-histamine receptors [43]. Additionally, in some circumstances, chlorpromazine exerts suppressive action on the immune response [16].

Currently, the spectrum of medications for the treatment of schizophrenia has been expanded with the introduction of atypical, new-generation drugs with fewer side effects. Given their impact on DA activity and affinity for various other receptors [44,45], these antipsychotics seem to be more effective in the treatment of negative symptoms and cognitive impairment than the typical compounds [46].

Quetiapine is a second-generation atypical antipsychotic that acts as an antagonist on multiple pathways, including DA transmission, 5-HT_2A_, 5-HT_2B_, 5-HT_2C_, α_1_- and α_2_-adrenergic as well as H_1_-histamine receptors [47,48]. Additionally, this drug binds to the 5-HT_1A_ receptor as a partial agonist [48]. It has also been suggested that quetiapine affects microglial activation and mitigates neuroinflammation [49,50,51].

An example of a novel atypical antipsychotic of the third generation is aripiprazole, which acts as a partial agonist of D_2_, D_3_, D_4_, 5-HT_1A_ and 5-HT_2C_; an inverse agonist of 5-HT_2B_; and an antagonist of 5-HT_2A_ and 5-HT_6_ receptors [52]. Furthermore, it has an affinity for α_1_-adrenergic and H_1_-histamine receptors [52]. Similar to quetiapine, some studies suggest that aripiprazole may exert an anti-inflammatory action via the inhibition of microglial activation [53].

Accordingly, in the present study, we sought to determine whether chronic treatment with chlorpromazine, quetiapine or aripiprazole influences schizophrenia-like behavioural disturbances at the level of sensorimotor gating in male offspring that were prenatally exposed to LPS. Simultaneously, we wanted to explore if the chosen antipsychotics display an impact on the neuroimmunological parameters in the brains of these animals, with a special focus on the CX3CL1-CX3CR1 and CD200-CD200R axes as well as factors related to microglial activity and immune response.

## 2. Materials and Methods

### 2.1. Animals

Wistar rats (Charles River, Sulzfeld, Germany) were housed under standard conditions with a room temperature of 23 °C, 12/12 h light/dark cycle (lights on at 6:00 am) and ad libitum access to water and food. After a period of acclimatisation, the pro-oestrus phase of the cycle was determined based on vaginal smears obtained daily from the females, which were subsequently placed with males for 12 hours. The presence of sperm in vaginal smears was assessed the next morning. Pregnant females (*n* = 36) were randomly divided into two equal groups, including (1) control and (2) LPS, for further proceedings.

All procedures were approved by the Animal Care Committee of the Maj Institute of Pharmacology, Polish Academy of Sciences, Cracow and complied with the International Council for Laboratory Animals and Guide for the Care and Use of Laboratory Animals (consent number: 236/2016). All possible efforts were made to minimise the number of animals used and their suffering. The investigators were not blinded to the experimental conditions. The numbers of animals included in each analysis are presented in the caption to the corresponding figure or table.

### 2.2. Prenatal Treatment with LPS

The administration of the bacterial endotoxin to pregnant rats was performed as previously reported [13,19]. LPS (from *Escherichia coli* 026:B6; Sigma-Aldrich, St. Louis, MO, USA) was dissolved to obtain a concentration of 2 mg/kg of body weight in 1 mL of saline. The solution was subcutaneously administered to pregnant females in the LPS group on alternate days starting from the 7th day of pregnancy between 9:00 and 10:00 am [16,20,21]. Control pregnant animals were submitted to the same treatment regimen with the corresponding volume (1 mL/kg) of saline. After delivery, the dams were allowed to rear their young until weaning (postnatal day 21, PND21). No differences in litter size and weight were observed between the control and LPS groups. In the present study, only male offspring were used; thus, they were transferred and housed in groups of five per cage under standard conditions until further procedures.

### 2.3. Behavioural Study—Prepulse Inhibition Test

The prepulse inhibition (PPI) test was performed two times: (1) when the offspring were at PND90 and (2) after chronic 14-day administration of antipsychotics. The PPI procedure was adopted with some modifications from our previously published studies [16,20,21]. The examinations were performed in eight sound-proof, ventilated startle cabinets (SR-LAB, San Diego Instruments, San Diego, CA, USA) with a single Plexiglas cylinder (inner diameter of 9 cm) attached to a moveable platform in each of them. A startle reflex was elicited in response to a sound generated by a high-frequency loudspeaker, producing both continuous 65 dB background noise and various acoustic stimuli, mounted inside each chamber. Platform movements resulting from the startle reaction were detected for each animal by a piezoelectric accelerometer during the 200 ms recording window. The data were digitised and used for subsequent calculations, where the maximum startle response (V_max_) and average startle amplitude (AVG) were further analysed.

Before the experiments, each chamber was individually calibrated using the external sensor to display a similar readout of the reference stimulus. After five minutes of habituation to the background noise, four types of acoustic stimuli were used in random order. Each trial consisted of either a single pulse alone [intensity 120 dB, duration 40 ms, (P)] or a pulse preceded by a prepulse at one out of three intensities [70, 75 and 80 dB; duration 20 ms; (PP)] applied 80 ms before a pulse. During each experimental session, 20 trials of each type were displayed with an interstimulus interval of 20 s. The V_max_ and AVG were recorded, and the percentage of PPI (%PPI) induced by each prepulse intensity was calculated as %PPI = [(P − PP)/P] × 100%.

### 2.4. Antipsychotic Drugs Administration

After the first PPI test at PND90, the offspring (both from the control and prenatally LPS-treated groups) were divided into three sets: (1) subjected to chlorpromazine administration (cohort 1), (2) treated with quetiapine (cohort 2), and (3) injected with aripiprazole (cohort 3). Then, each cohort was further split to finally form the following experimental groups: control + vehicle to chlorpromazine, control + chlorpromazine, LPS + vehicle to chlorpromazine, LPS + chlorpromazine, control + vehicle to quetiapine, control + quetiapine, LPS + vehicle to quetiapine, LPS + quetiapine, control + vehicle to aripiprazole, control + aripiprazole, LPS + vehicle to aripiprazole, and LPS + aripiprazole (Table 1).

Chlorpromazine (Sigma-Aldrich, St. Louis, MO, USA) was dissolved in 1 mL of saline to obtain a concentration of 10 mg/kg [54,55]. Quetiapine (Carbosynth, Berkshire, UK) was prepared as a 10 mg/kg solution in 0.8% acetic acid in 1 mL of saline (pH adjusted with 1 N NaOH) [56,57]. Aripiprazole (Carbosynth, Berkshire, UK) was dissolved in 5% dimethyl sulfoxide (BioShop, Burlington, ON, Canada) in 1 mL of saline to a concentration of 1 mg/kg [58,59].

Antipsychotic drugs were administered intraperitoneally once daily between 9:00 and 10:00 am for 14 days. The control groups for each drug received an intraperitoneal injection of the appropriate vehicle in the corresponding volume (1 mL/kg) and regimen. Twenty-four hours after the last dose, the animals underwent the PPI test again.

### 2.5. Biochemical Study

#### 2.5.1. Tissue Collection and Preparation

The frontal cortices and hippocampi were collected from the animals the day following the last behavioural examination. The tissues were dissected on an ice-cold glass plate and stored at −80 °C until further processing.

Samples intended for analyses with enzyme-linked immunosorbent assay (ELISA) were homogenised by Tissue Lyser II (Qiagen Inc., Valencia, CA, USA) in RIPA lysis buffer enriched with protease inhibitor cocktail, phosphatase inhibitor cocktail, 1 mM sodium orthovanadate and 1 mM phenylmethanesulfonyl fluoride (all from Sigma-Aldrich, St. Louis, MO, USA). The protein concentration in the prepared samples was evaluated using a Pierce^TM^ BCA Protein Assay Kit (Thermo Fisher, Rockford, IL, USA) according to the manufacturer’s instructions. Bovine serum albumin from the kit was applied as a standard, and the absorbance for each sample was measured at a wavelength of 562 nm in a Tecan Infinite 200 Pro spectrophotometer (Tecan, Mannedorf, Germany).

In order to prepare probes for quantitative real-time polymerase chain reaction (qRT-PCR), the tissues were initially homogenised by Tissue Lyser II (Qiagen Inc., Valencia, CA, USA) with an appropriate volume of lysis buffer supplied with a Total RNA Mini Plus kit (A&A Biotechnology, Gdynia, Poland). Further, the manual provided with the set was followed to obtain total RNA from the frontal cortices and/or hippocampi of the rats. Immediately after extraction, the RNA concentration was determined by a NanoDrop Spectrophotometer (ND/1000 UV/Vis, Thermo Fisher NanoDrop, Waltham, MA, USA).

#### 2.5.2. Quantitative Real-Time Polymerase Chain Reaction

The synthesis of complementary DNA (cDNA) from equal amounts of RNA (1 μg) via reverse transcription was performed using the NG dART RT kit (EURx, Gdańsk, Poland). The cDNA was amplified with a FastStart Universal Probe Master (Rox) kit (Roche, Basel, Switzerland) and TaqMan probes (Life Technologies, Carlsbad, CA, USA) for the genes: *Cx3cl1* (Rn00593186_m1), *Cx3cr1* (Rn00591798_m1), *Cd200* (Rn01646320_m1), *Cd200r* (Rn00576646_m1), *Cd40* (Rn01423583_m1), *Cd68* (Rn01495634_g1), *Arg1* (Rn00691090_m1), *Igf-1* (Rn00710306_m1) and, as the reference, *Gapdh* (Rn01775763_g1). The PCR products were generated in mixtures consisting of cDNA used as the PCR template (1 μL), TaqMan forward and reverse primers (1 μL), 1× FastStart Universal Probe Master (Rox) mix containing 250 nM of hydrolysis probe labelled with the fluorescent reporter dye (fluorescein) at the 5′-end and a quenching dye at the 3′-end (10 μL), and finally the remainder of PCR grade distilled water to a total volume of 20 μL. The thermocycling conditions were as follows: initial denaturation at 95 °C for 10 minutes, 45 cycles of denaturation at 95 °C for 15 s, annealing at 60 °C for 1 minute and extension at 50 °C for 2 minutes. The threshold value (C_t_) for each sample was set in the exponential phase of PCR, and the ∆∆C_t_ method was used for the data analysis.

#### 2.5.3. Enzyme-Linked Immunosorbent Assay

IL-1β, TNF-α (both from Thermo Fisher Scientific, Waltham, MA, USA), IL-4, IL-6 and IL-10 (all three from BD Biosciences, San Diego, CA, USA) protein levels in the frontal cortices of the rats were established using commercially available ELISA kits following the manufacturer’s instructions.

### 2.6. Statistical Data Analysis

Statistical analysis of the data was performed using Statistica 13.0 software (StatSoft, Palo Alto, CA, USA). The data from the PPI test are demonstrated as the mean ± SEM. The results from qRT-PCR studies are displayed as the average fold change ± SEM, whereas those from ELISA experiments are presented as the mean ± SEM. Comparisons of variables between groups were performed using two-way analysis of variance (ANOVA) with Duncan’s post hoc except for the PPI test at PND90 when Student’s *t* test was applied. The results were considered statistically significant when the *p* value was less than 0.05. When applicable, statistical outliers were identified using Grubbs’ test. All graphs were prepared with GraphPad Prism 7 software (San Diego, CA, USA).

## 3. Results

### 3.1. Prepulse Inhibition of the Acoustic Startle Response

PPI is a measure of sensorimotor gating [60], the impairment of which has been widely reported in patients with schizophrenia [61,62,63]. In rodents, experimentally induced deficits in PPI are employed as an endophenotype to reflect this basic schizophrenia-like behaviour and to study the mechanisms underlying attentional and cognitive disturbances [22,64,65].

**Figure 1:**
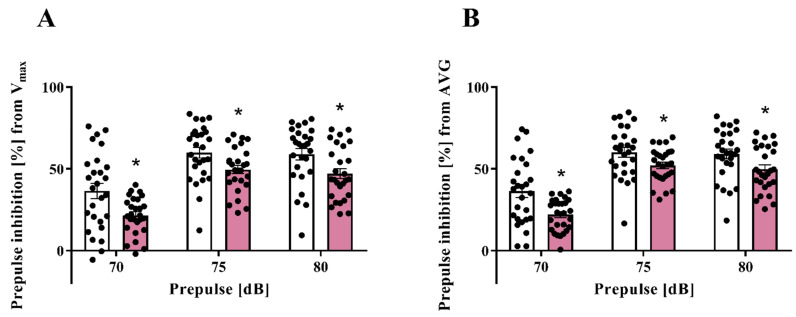
Representative prepulse inhibition (PPI) of the acoustic startle response in the control and prenatally LPS-exposed offspring at PND90 (*n* = 27 in each group). The results are presented as the means of the percentage of PPI (%PPI) calculated from the maximum startle response (V_max_) (**A**) and average startle amplitude (AVG) (**B**) induced by each prepulse intensity ± SEM. * *p* < 0.05 vs. control group.

Consistent with previously published data [13,16,17,66], the treatment of female rats with LPS during pregnancy generated a significant inhibition of sensorimotor gating in the adult offspring both in terms of V_max_ (Figure 1A) and AVG (Figure 1B). As exemplified by the second cohort, this phenomenon was evidenced by the disruption of PPI for all tested prepulse intensities of V_max_: 70 (36.52 ± 4.54 control vs. 21.60 ± 2.18 LPS, F_(1,52)_ = 4.32, *p* = 0.0046), 75 (59.92 ± 3.27 control vs. 49.60 ± 2.59 LPS, F_(1,52)_ = 1.59, *p* = 0.0167) and 80 (58.92 ± 3.39 control vs. 47.15 ± 3.13 LPS, F_(1,52)_ = 1.17, *p* = 0.0138) dB as well as AVG: 70 (36.34 ± 3.92 control vs. 22.01 ± 1.89 LPS, F_(1,52)_ = 4.29, *p* = 0.0018), 75 (60.17 ± 2.98 control vs. 52.14 ± 2.00 LPS, F_(1,52)_ = 2.22, *p* = 0.0298) and 80 (59.11 ± 2.97 control vs. 49.81 ± 2.66 LPS, F_(1,52)_ = 1.25, *p* = 0.0238) dB (Figure 1).

**Figure 2:**
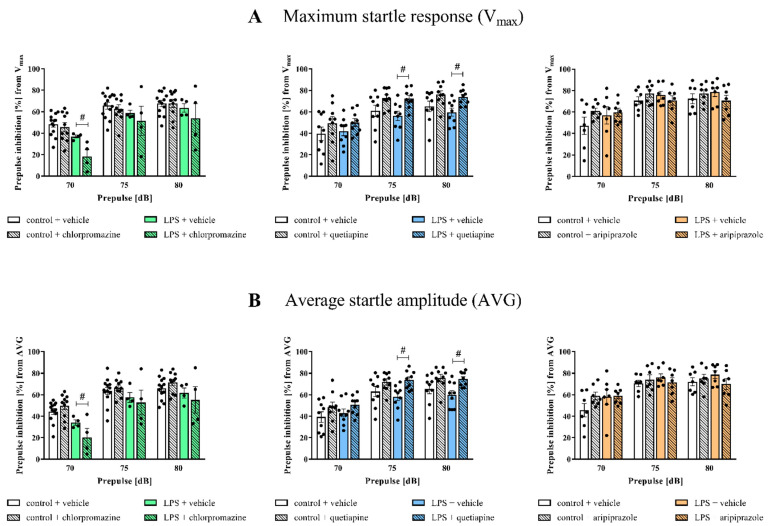
The impact of 14-day intraperitoneal administration of chlorpromazine, quetiapine or aripiprazole on the prepulse inhibition (PPI) of the acoustic startle response in the control and prenatally LPS-exposed offspring in adulthood. The numbers of animals in the cohorts were as follows: *n* = 4–13 (chlorpromazine), *n* = 9 (quetiapine) and *n* = 7 (aripiprazole) in each group. The results are presented as the means of the percentage of PPI (%PPI) calculated from the maximum startle response (V_max_) (**A**) and average startle amplitude (AVG) (**B**) induced by each prepulse intensity ± SEM. # *p* < 0.05 vs. LPS + vehicle group.

Next, to examine the ability of chlorpromazine, quetiapine and aripiprazole to normalise the observed PPI deficits in the applied experimental conditions, we subjected the animals to injections of these antipsychotic drugs for 14 days (Figure 2).

Subsequent behavioural examination revealed that chlorpromazine reduced the PPI of both V_max_ (36.98 ± 1.39 LPS + vehicle vs. 18.45 ± 6.27 LPS + chlorpromazine, F_(1,27)_ = 5.28, *p* = 0.0085) and AVG (34.09 ± 2.36 LPS + vehicle vs. 20.39 ± 8.31 LPS, F_(1,27)_ = 0.35, *p* = 0.0379) for 70 dB in the LPS-exposed offspring (Figure 2).

In terms of quetiapine, the drug displayed a beneficial impact on the examined behaviour, manifesting as an alleviated PPI response in the rats. The outcome for the LPS + quetiapine group was significantly different from the LPS + vehicle offspring both at the level of V_max_ for 75 (56.33 ± 4.26 LPS + vehicle vs. 72.52 ± 2.97 LPS + quetiapine, F_(1,32)_ = 12.42, *p* = 0.0098) and 80 (59.63 ± 3.73 LPS + vehicle vs. 73.76 ± 2.43 LPS + quetiapine, F_(1,32)_ = 11.07, *p* = 0.0157) dB and AVG for 75 (58.17 ± 3.70 LPS + vehicle vs. 73.74 ± 2.84 LPS + quetiapine, F_(1,32)_ = 11.25, *p* = 0.0082) and 80 (59.87 ± 3.99 LPS + vehicle vs. 74.72 ± 2.27 LPS + quetiapine, F_(1,32)_ = 12.16, *p* = 0.0089) dB prepulse intensities. In the control animals, the administration of quetiapine resulted in an increasing tendency in V_max_ for 75 (61.11 ± 5.29 control + vehicle vs. 73.06 ± 2.96 control + quetiapine, F_(1,32)_ = 12.42, *p* = 0.0527) and 80 (65.24 ± 4.92 control + vehicle vs. 76.03 ± 3.46 control + quetiapine, F_(1,32)_ = 11.07, *p* = 0.0618) dB and AVG for 75 (62.99 ± 4.82 control + vehicle vs. 71.88 ± 2.87 control + quetiapine, F_(1,32)_ = 11.25, *p* = 0.0945) and 80 (65.35 ± 4.49 control + vehicle vs. 75.71 ± 3.33 control + quetiapine, F_(1,32)_ = 12.16, *p* = 0.0631) dB (Figure 2).

The results of the PPI test conducted for the cohort of rats treated with aripiprazole showed no effect of the drug on V_max_ or AVG for any of the evaluated prepulse levels (Figure 2).

### 3.2. Cx3cl1, Cx3cr1, Cd200 and Cd200r mRNA Expression in the Frontal Cortices of the Offspring

In the first set of biochemical experiments, we assessed the mRNA expression of the systems controlling neuron–microglia interactions in the frontal cortices of the prenatally LPS-exposed offspring and the influence of chronic chlorpromazine, quetiapine and aripiprazole treatment in adulthood on these factors (Table 2).

An analysis of the cortical homogenates of the LPS-subjected animals from the first cohort demonstrated an elevation of the mRNA levels of *Cx3cl1* (1.38 ± 0.57 control + vehicle vs. 5.27 ± 1.20 LPS + vehicle, F_(1,16)_ = 28.56, *p* = 0.0079), *Cx3cr1* (1.28 ± 0.46 control + vehicle vs. 5.39 ± 1.83 LPS + vehicle, F_(1,16)_ = 23.78, *p* = 0.0248), *Cd200* (1.28 ± 0.43 control + vehicle vs. 4.34 ± 1.23 LPS + vehicle, F_(1,16)_ = 13.60, *p* = 0.0246) and *Cd200r* (1.33 ± 0.46 control + vehicle vs. 13.24 ± 4.58 LPS + vehicle, F_(1,16)_ = 23.91, *p* = 0.0026) compared to the appropriate control group. Regarding the impact of chlorpromazine on the analysed parameters, we observed only an increasing tendency in *Cx3cr1* expression (5.39 ± 1.83 LPS + vehicle vs. LPS + chlorpromazine, F_(1,16)_ = 1.89, *p* = 0.0866) (Table 2A).

A study of samples obtained from the second cohort showed upregulation of *Cd200r* level (1.12 ± 0.22 control + vehicle vs. 2.95 ± 0.65 LPS + vehicle, F_(1,19)_ = 12.93, *p* = 0.0202) and an increasing trend in the mRNA expression of *Cx3cr1* (1.01 ± 0.04 control + vehicle vs. 1.51 ± 0.23 LPS + vehicle, F_(1,32)_ = 3.97, *p* = 0.0762) in the frontal cortices of the rats prenatally subjected to LPS. We did not observe an effect of quetiapine injections on any of the investigated factors (Table 2B).

In the third cohort, prenatal exposure to LPS resulted in significantly raised *Cd200r* (1.10 ± 0.22 control + vehicle vs. 2.61 ± 0.53 LPS + vehicle, F_(1,20)_ = 5.48, *p* = 0.0215) mRNA level. Aripiprazole administration did not influence gene expression in the frontal cortices of the animals in any of the examined groups (Table 2C).

### 3.3. Cd40, Cd68, Arg1 and Igf-1 mRNA Expression in the Frontal Cortices of the Offspring

The axes controlling neuron–microglia communication (CX3CL1-CX3CR1 and CD200-CD200R) are highly involved in the regulation of microglial activation [25,26]. Since we found that the mRNA levels of these genes were to some extent affected by prenatal treatment with LPS but not significantly influenced by the antipsychotics, in the next step, we wanted to determine the expression of the microglia-related pro- (*Cd40* and *Cd68*) and anti-inflammatory (*Arg1* and *Igf-1*) markers (Table 3).

qRT-PCR revealed no significant differences in any measured cortical parameters in the offspring from any experimental group of the first cohort (Table 3A).

As per the gene expression in the frontal cortices of the rats from the second cohort, we detected a higher mRNA level of *Cd68* (0.87 ± 0.05 control + vehicle vs. 1.18 ± 0.11 LPS + vehicle, F_(1,31)_ = 5.91, *p* = 0.0222) and an increasing trend in *Arg1* (1.12 ± 0.23 control + vehicle vs. 2.04 ± 0.52 LPS + vehicle, F_(1,20)_ = 14.71, *p* = 0.0952) level due to prenatal LPS exposure. Subsequent 14-day administration of quetiapine normalized *Cd68* (1.18 ± 0.11 LPS + vehicle vs. 0.86 ± 0.09 LPS + quetiapine, F_(1,31)_ = 6.20, *p* = 0.0259) expression (Table 3B).

Considering the animals from the third cohort, the LPS-treated group was characterized by upregulated expression of *Igf-1* (1.01 ± 0.05 control + vehicle vs. 1.27 ± 0.08 LPS + vehicle, F_(1,19)_ = 14.38, *p* = 0.0103). We did not note any impact of aripiprazole injections on the evaluated microglia-associated markers (Table 3C).

### 3.4. Levels of Pro- and Anti-Inflammatory Cytokines in the Frontal Cortices of the Offspring

**Figure 3:**
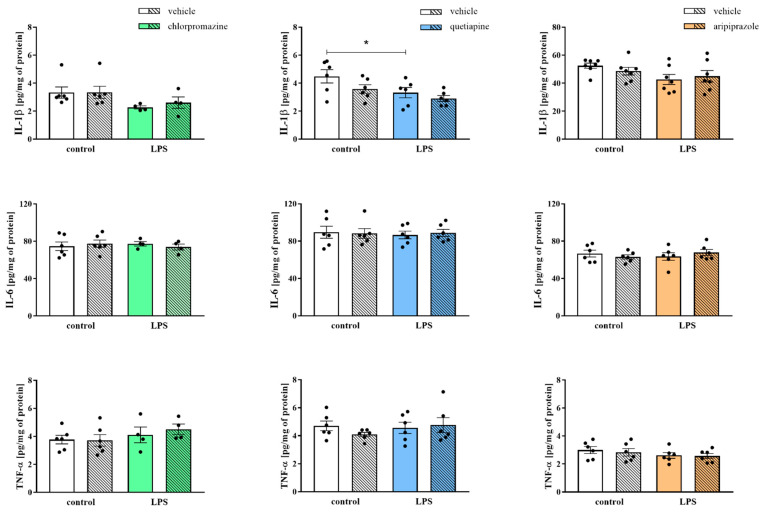
The impact of prenatal exposure to LPS and subsequent chronic treatment with chlorpromazine (*n* = 4–6 in each group), quetiapine (*n* = 6 in each group) or aripiprazole (*n* = 6–7 in each group) on the levels of the proinflammatory proteins (IL-1β, IL-6 and TNF-α) in the frontal cortices of the offspring. The results are presented as the mean ± SEM. * *p* < 0.05 vs. control + vehicle.

**Figure 4:**
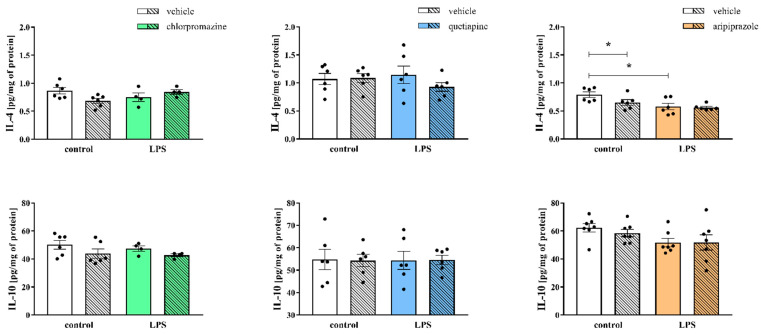
The impact of prenatal exposure to LPS and subsequent chronic treatment with chlorpromazine (*n* = 4–6 in each group), quetiapine (*n* = 6 in each group) or aripiprazole (*n* = 6–7 in each group) on the levels of the anti-inflammatory proteins (IL-4 and IL-10) in the frontal cortices of the offspring. The results are presented as the mean ± SEM. * *p* < 0.05 vs. control + vehicle.

Concurrently, we determined the protein levels of selected pro- (IL-1β, IL-6, TNF-α) and anti-inflammatory (IL-4, IL-10) factors that are crucial in the immune response and microglial activation in the frontal cortices of the prenatally LPS-subjected animals and the effect of the administration of antipsychotics in adulthood on these parameters (Figure 3, Figure 4).

In the first cohort, we observed a decreasing tendency in IL-1β (3.33 ± 0.40 control + vehicle vs. 2.27 ± 0.11 LPS + vehicle, F_(1,16)_ = 4.90, *p* = 0.0975) level resulting from LPS exposure. ELISA analysis presented no significant differences in any of the quantified cortical protein levels after chlorpromazine injections (Figure 3).

Regarding the results obtained from the second cohort, we found a reduction in IL-1β (4.49 ± 0.48 control + vehicle vs. 3.33 ± 0.37 LPS + vehicle, F_(1,20)_ = 6.82, *p* = 0.0375) level in the frontal cortices of the LPS-treated offspring. Quetiapine administration resulted in trend of reduced IL-1β (4.49 ± 0.48 control + vehicle vs. 3.58 ± 0.30 control + quetiapine, F_(1,20)_ = 3.54, *p* = 0.0840) level in control rats (Figure 3).

As in the case of the third cohort, prenatal contact with LPS led to a decreasing tendency in the cortical level of IL-1β (52.46 ± 1.90 control + vehicle vs. 42.62 ± 3.59 LPS + vehicle, F_(1,24)_ = 4.46, *p* = 0.0567). No significant impact of aripiprazole on the examined proteins was detected in either the control or LPS-exposed animals (Figure 3).

Along with these results, we did not observe any changes in the levels of IL-6 and TNF-α either after prenatal exposure to LPS or chlorpromazine, quetiapine or aripiprazole medication in any of the rat cohorts (Figure 3).

Among the tested markers of the anti-inflammatory profile, we found a decreasing tendency in IL-4 (0.87 ± 0.06 control + vehicle vs. 0.68 ± 0.04 control + chlorpromazine, F_(1,16)_ = 0.58, *p* = 0.0503) level after chlorpromazine treatment in the control group from the first cohort. Statistical analysis exhibited no effect of either LPS or the drug on IL-4 and IL-10 protein levels (Figure 4).

The ELISA results for the cortical homogenates of the offspring from the second cohort revealed no alterations in the estimated cytokines levels after prenatal LPS exposure or the later quetiapine injections (Figure 4).

In the frontal cortices of the rats prenatally subjected to LPS from the third cohort, we showed a decline in IL-4 (0.79 ± 0.05 control + vehicle vs. 0.58 ± 0.06 LPS + vehicle, F_(1,20)_ = 10.66, *p* = 0.0056) level and a decreasing tendency in IL-10 (62.34 ± 2.99 control + vehicle vs. 51.75 ± 3.02 LPS + vehicle, F_(1,24)_ = 5.43, *p* = 0.0745) level compared to control animals. Simultaneously, the cortical level of IL-4 (0.79 ± 0.05 control + vehicle vs. 0.65 ± 0.05 control + aripiprazole, F_(1,20)_ = 2.86, *p* = 0.0467) was reduced after aripiprazole treatment in the control group (Figure 4).

### 3.5. Cx3cl1, Cx3cr1, Cd200, Cd200r, Cd40, Cd68, Arg1 and Igf-1 mRNA Expression in the Hippocampi of the Offspring

Having found that quetiapine exhibited the most prominent effect on the PPI from the selected antipsychotics, but the impact of this drug on the gene expression of measured parameters in the frontal cortex was minimal, we additionally analysed the mRNA levels of *Cx3cl1*, *Cx3cr1*, *Cd200*, *Cd200r*, *Cd40*, *Cd68*, *Arg1* and *Igf-1* in the hippocampi of the offspring from the second cohort (Table 4).

The hippocampal gene expression of the chosen factors was not altered by exposure to LPS during the prenatal period, and the administration of quetiapine did not affect these parameters (Table 4A,B). However, analyses of the homogenates generated from the control rats showed a growing tendency in the levels of *Cx3cl1* (1.02 ± 0.08 control + vehicle vs. 1.56 ± 0.27 control + quetiapine, F_(1,28)_ = 4.61, *p* = 0.0896) and *Cx3cr1* (1.10 ± 0.18 control + vehicle vs. 1.91 ± 0.37 control + quetiapine, F_(1,28)_ = 3.84, *p* = 0.0730) after chronic drug delivery (Table 4A).

## 4. Discussion

The present study aimed to investigate the potential effect of chronic antipsychotic drugs on behavioural deficits observed in adult offspring prenatally treated with LPS as well as the cortical and/or hippocampal expression of the *Cx3cl1*-*Cx3cr1* and *Cd200*-*Cd200r* axes, which are essential in the determination of microglial profile and immune response.

We confirmed that MIA, induced by LPS treatment, leads to behavioural disturbances expressed as deficits in sensorimotor gating in adult offspring. Furthermore, among the tested antipsychotic drugs, quetiapine alleviated the altered behaviour and attenuated MIA-upregulated expression of *Cd68* in the frontal cortex. Furthermore, quetiapine moderately modulated the hippocampal mRNA levels of the *Cx3cl1*-*Cx3cr1* axis.

As previously described [13,16,19,20,21], our research was conducted in a neurodevelopmental rat model of schizophrenia, the principle of which involves the administration of bacterial endotoxin (LPS) to pregnant females throughout pregnancy (see details in the Materials and Methods section). This experimental approach has demonstrated validity at the face [13,67], predictive [16,21] and construct [19,20,67] levels. Prenatal exposure to LPS induces behavioural deficits expressed as disturbances in exploration [20,67], spontaneous and amphetamine-stimulated locomotor activity changes [21], social interaction deficits [67] and the presence of anxiety behaviour [19]. It has also been reported that the antenatal administration of LPS generates deficits in sensorimotor gating in an age-dependent manner [13,20]. Herein, we again confirmed this phenomenon showing that LPS treatment during pregnancy leads to behavioural disturbances expressed as an altered PPI in adult offspring. It is particularly important in terms of schizophrenia symptoms given that, despite being also observed in several other neuropsychiatric disorders [68], impaired sensorimotor gating is considered one of the behavioural hallmarks of this condition [69]. This feature reflects the brain’s ability to filter out irrelevant information before it reaches high levels of conscious processing [70] and can be displayed as PPI, which occurs when a weak, subthreshold stimulus presented prior to an intense startling stimulus inhibits the startle response [71].

In our study, PPI was applied not only as a useful tool in evaluating the impact of environmental risk factors (in the form of prenatal exposure to LPS) during development but also in the context of its possible pharmacological modulation by chronic treatment with antipsychotics. For this purpose, in the experiments described here, we introduced three antipsychotics that strongly varied in pharmacological action profiles, specifically chlorpromazine, quetiapine and aripiprazole.

Chlorpromazine belongs to the group of phenothiazines that are primarily used for the treatment of schizophrenia and acts mainly in subcortical structures of the brain [72]. It is not a selective drug, as it interacts with DA (mainly with D_2_ receptors), noradrenergic, glutamatergic (GLU), 5-HT and histaminergic systems and with some intracellular processes, such as inhibition of nitric oxide synthase or the activity of calmodulin and protein kinase C [73,74]. Notably, PPI deficits can be observed in rats treated with psychotomimetic agents, including DA agonists and GLU antagonists [75]. DA agonist-induced changes in PPI are reversed by both typical and atypical antipsychotics, whereas those generated by GLU antagonists seem to be alleviated only by atypical antipsychotics [75]. However, regarding chlorpromazine, available data on its impact on PPI still appear to heavily depend on the model applied in the research [76,77,78]. In the present study, we did not observe a favourable effect of this drug on the PPI deficit, which corresponds with some previously published reports [16,21]. Contrary, for the weakest prepulse (70 dB), transitional PPI reduction was found in the MIA model after chlorpromazine administration. A similar effect was not observed in control animals as well as for other prepulse intensities (75 and 80 dB), which suggests that it was most likely unrelated to the effect of this drug on motor functions. Since chlorpromazine is not a selective compound, the modulatory impact of MIA on the profile and sensitivity of multiple receptors affected by this drug (including noradrenergic and histaminergic) should not be excluded. However, this phenomenon requires further research. Yet, in the LPS-produced MIA model, chlorpromazine showed other beneficial effects, including reduction of amphetamine-induced hyperactivity, normalisation of the HPA axis and deficits in the level of glucocorticoid receptors in the hippocampus as well as balancing MIA-evoked changes in the peripheral immune response (including cytokines IL-1β, TNF-α and IL-2) [16,21].

Support for the application of atypical antipsychotics in schizophrenia therapy comes, among others, from their efficacy against negative symptoms and, to some extent, impaired cognitive functions [79,80,81]. Nevertheless, in the present study, chronic 14-day treatment with aripiprazole showed no influence on the PPI deficit in the LPS-subjected offspring. Some data demonstrated that aripiprazole ameliorates behavioural disturbances in mice following prenatal polyinosinic:polycytidylic acid (poly I:C) treatment [82]. Interestingly, the antipsychotic effect of this drug in the MK-801-induced rat model of schizophrenia seems to be related to its action both through 5-HT_1A_ and D_1_ receptors, as cotreatment with antidepressants potentiates the pro-cognitive effects of aripiprazole [83]. Clinical studies have reported that this drug significantly ameliorates a broad range of symptoms in schizophrenia and schizoaffective disorders over a short-term period [84,85]. Fejgin and colleagues suggested that the preclinical efficacy of short-term aripiprazole treatment in improving the impairment of prepulse inhibition may rely on its partial agonism leading to DA stabilising effects [86]. This action can be mediated differently by the dose of the drug and is highly dependent on the affected brain structure [87]. Therefore, the lack of favourable aripiprazole influence in the PPI test may result from the specificity of the MIA model employed and requires further investigation using a different schedule of experiments or drug administration (time, dose, etc.).

The most important finding of our multicohort study is the observation that quetiapine was able to normalise the PPI disturbances in adult male offspring in the MIA model. As presented in the literature, this atypical antipsychotic has shown efficacy in treating positive and negative symptoms as well as cognitive impairment in schizophrenia patients [88]. Moreover, consistent with our data, quetiapine attenuated schizophrenia-like behaviours, including not only sensorimotor gating deficits but also hyperactivity and memory impairment, in the MK-801-treated mouse model [88]. Therefore, the present research reaffirmed the face and construct validity of the use of MIA in modelling schizophrenia-like symptoms and the predictive validity of quetiapine administration.

The complete picture of antipsychotic drug action in the treatment of schizophrenia has not been fully elucidated. In addition to the affinity for neurotransmitter receptors [89,90], their potential modulatory role on inflammatory processes in the emergence of schizophrenia-like symptoms is strongly postulated [91]. Growing evidence suggests that proper neuron–microglia interactions are of key importance for the control of the immune response, whereas dysfunction of this dynamic crosstalk leads to microglial activation and exaggerated inflammation [11,28,92,93]. Our data strongly suggest that changes in the CX3CL1-CX3CR1 and CD200-CD200R axes observed in the MIA model can be crucial in the development of behavioural disturbances [13]. Most malfunctions were observed in the brains of 7-day-old offspring, albeit some were long-lasting and present in adulthood [13]. Therefore, the question arises whether antipsychotics, specifically chlorpromazine, with high immunosuppressive potential [21,94], as well as atypical drugs with modulatory properties on glial activity [50,51,95], can affect the mentioned ligand-receptor communications and consequently, microglial phenotype.

The most striking finding from the present study was the upregulation of *Cd200r* expression in the frontal cortex of the animals in the MIA model. Additionally, we demonstrated variations in the ligand (*Cd200*) as well as the *Cx3cl1*-*Cx3cr1* axis levels. Chronic treatment with antipsychotics did not significantly affect these modifications, although quetiapine showed a positive trend for an increase in *Cx3cl1*-*Cx3cr1* expression in the hippocampus, in which deficits in neurotransmission (e.g., GABAergic) are particularly strongly expressed in male offspring in the MIA model [67]. To the best of our knowledge, the above-described observations are the first to address this issue and are thereby difficult to interpret.

As it is commonly accepted that both axes play a crucial role in the regulation of microglial activation, in the next set of experiments, we analysed the expression of selected pro- (*Cd40*, *Cd68*) and anti- (*Arg1*, *Igf-1*) inflammatory markers after administration of antipsychotic drugs in the MIA model. The only significant observation was quetiapine’s ability to lower elevated *Cd68* expression in the frontal cortex of adult offspring prenatally treated with LPS. CD68 is a member of a growing family of haematopoietic mucin-like molecules that are present in macrophages/microglia [96]. It is not only a marker of cell proliferation but also carries the potential to label lysosomal and endosomal transmembrane glycoproteins of microglia, indicating phagocytic activity [97]. Previous literature looking at CD68 expression in control and schizophrenia cases has reported ambiguous outcomes [98,99].

Microglial dysfunction and neuroinflammation are thought to contribute to the pathogenesis of schizophrenia [100,101]. Nevertheless, given that schizophrenia is not a progressive but a chronic condition, it is possible that changes in glial dynamics may be more pronounced closer to disease onset, and these disturbances would have subsided later in time. In young 7-day-old offspring that were prenatally exposed to LPS, we found not only upregulation of cortical *Cd40* and IBA1 levels but also various changes in the hippocampus, for example, enhanced mRNA expression of *Il-1β*, *Tnf-α*, *Arg1*, *Tgf-β* and *Il-10* [13]. Hence, microglial activation is not a sustained event within a schizophrenia-like model [102], which may, at least in part, explain the slight changes observed in the present study.

Our results contradict some observations in other experimental models, in which antipsychotics have been shown to modulate glial reactivity. It has been found that the anti-inflammatory properties (suppression of IL-1β and IL-2 from microglial cells activated with LPS) of chlorpromazine may be partly derived from their ability to inhibit microglial proton currents [103]. However, it should be noted that chronic treatment with phenothiazines itself might have pro-oxidant effects because chlorpromazine metabolites have been shown to undergo auto-oxidation, generating hydrogen peroxide [104,105]. Simultaneously, aripiprazole not only inhibits interferon-γ-induced microglial activation and superoxide generation [53,106] but also limits oligodendrocyte damage caused by activated microglial cells [107]. In RAW264.7 cells, aripiprazole suppresses the expression of proinflammatory genes, such as cyclooxygenase-2, inducible NO synthase and TNF-α through inhibition of both the AP-1 and NF-κB pathways [95]. Interestingly, the antipsychotic activity of aripiprazole in a schizophrenia-like poly I:C-induced model may be related to the limitation of microglial inflammatory reactions as well as TRPM7 receptor suppression [108]. Regarding quetiapine, it inhibits NO generation by activated microglia in vitro [49]. Moreover, this drug reduces the microglial number in the hippocampus and attenuates Aβ1-42-induced glial activation in APP/PS1 transgenic mice [51,109]. However, in the context of the present study, of crucial importance seems to be the observation that the efficiency of quetiapine’s anti-inflammatory action appears to be dependent on the “previous” inflammatory activation state of cells. For example, in nonactivated conditions, this drug triggered a consistent inflammatory response (including a high level of NO, proinflammatory gene expression and diminished level of IL-10). In contrast, after stimulation, the effects of quetiapine revealed an anti-inflammatory profile [110]. This finding points to its immunomodulatory potential, which was also observed in clinical studies in first-episode psychosis patients [111]. Therefore, we hypothesise that the limited impact of quetiapine on the neuron–microglia axes and, consequently, microglial activation may be related to the absence of their activation in offspring prenatally treated with LPS.

Finally, to further characterise the influence of antipsychotics on the immune response in the MIA model, we evaluated their potential impact on cytokine release. However, the consequence of MIA on the levels of proinflammatory cytokines (IL-6, TNF-α, IL-1β) in the frontal cortex of adult rats was marginal. In addition, we did not find an effect of chronic administration of quetiapine and other drugs on the levels of these proteins.

Recently, a retrospective analysis revealed elevated peripheral and/or cerebrospinal fluid IL-6 levels in schizophrenic patients [112]; however, there are also reports of reduced production of this cytokine [8]. Simultaneously, other research does not find a strict positive correlation between the severity of positive symptoms, cognitive deficits and enhanced levels of proinflammatory cytokines, such as IL-6, IL-1β and TNF-α [113,114,115]. Moreover, inflammation leads to structural brain changes via activation of microglia and/or astrocytic dysfunction, which determine the alterations in proinflammatory factors depending on the area of the brain [116]. It thus follows that the level of cytokines and the effect of the administered drugs are largely associated not only with the “previous” status of cell activation but also with many other factors, such as the experimental procedure, the age of animals and/or patients, the course of illness and the pharmacotherapy and its duration, which undoubtedly complicates the ability to obtain unambiguous results [117]. An alternative (but not mutually exclusive) mechanism of the lack of proinflammatory status in the MIA model may relate to the upregulation of the sIL-1Ra protein level, which can block the inflammatory effects typically associated with IL-1 signalling [118]. Similarly, a significant increase in sTNFR levels observed in schizophrenic patients [119] can potentially limit proinflammatory processes [120,121].

Regarding IL-10 level, we did not detect changes in the frontal cortices of the adult offspring either from the MIA group or after the administration of antipsychotics. Although quetiapine and its metabolite norquetiapine appear to have an anti-inflammatory action, in particular on IL-10 and IFN-γ following acute LPS challenge in serum and brain, this effect did not translate into behavioural changes [122]. Additionally, there was no evidence that the modulation of these changes influenced the levels of other cytokines, which indicates a rather partial immunomodulatory potential of both drugs in the LPS-induced model [122].

The involvement of IL-4 in the pathogenesis of schizophrenia has been widely investigated, yet published data remain highly heterogeneous [123,124]. In the present study, we showed a reduction in the IL-4 level in the group of MIA animals. This observation corresponds with several reports presenting significantly lower IL-4 levels in patients suffering from schizophrenia compared to healthy controls [125,126,127]. Furthermore, in our conditions, the detected change in cortical IL-4 production was not affected by exposure to any of the examined drugs. A few meta-analyses have confirmed no disturbances in IL-4 levels after treatment with antipsychotics [128,129]. However, in line with the inconsistency related to basal IL-4 levels in schizophrenia, there is also contradictory evidence concerning the effects of pharmaceuticals on this cytokine. Some findings revealed that treatment with typical or atypical antipsychotics or a combination of them led to decreased levels of IL-4 [130,131,132]. Herein, we noted a decline in IL-4 expression after aripiprazole treatment in the control offspring. Romeo et al. described a similar trend in their meta-analysis referring to the administration of this drug [129]. Nevertheless, the role of IL-4 in schizophrenia-related disturbances remains ambiguous and undoubtedly requires further investigation.

Overall, the present study accentuates the complexity of the possible link between chronic treatment with antipsychotics and behavioural schizophrenia-like disturbances as well as the axes controlling neuron–microglia communication and immune response in prenatally LPS-subjected adult male rats. Given that our data failed to establish an unequivocal association between these aspects, we feel it is reasonable to conclude that the beneficial impact of quetiapine on the onset of PPI malfunction in adulthood was not primarily the result of changes either in the *Cx3cl1*-*Cx3cr1* and *Cd200*-*Cd200r* axes or the pronounced modulation of microglia activation by this drug. However, it is necessary to mention once again that such a relationship cannot be fully excluded due to the double-faced immunomodulatory mechanism of quetiapine action. Nevertheless, our study provided the basis for further research to identify the CX3CL1-CX3CR1 and/or CD200-CD200R axes as new potential targets for antipsychotics in a model of schizophrenia-like behavioural disturbances.

## Figures and Tables

**Table 1:** The groups of the offspring generated and subsequently subjected to the administration of the antipsychotic drugs. The number of animals in each group is reported.

Cohort 1	Cohort 2	Cohort 3
chlorpromazine	quetiapine	aripiprazole
control + vehicle to chlorpromazine (*n* = 13)	control + vehicle to quetiapine (*n* = 9)	control + vehicle to aripiprazole (*n* = 7)
control + chlorpromazine (*n* = 10)	control + quetiapine (*n* = 9)	control + aripiprazole (*n* = 7)
LPS + vehicle to chlorpromazine (*n* = 4)	LPS + vehicle to quetiapine (*n* = 9)	LPS + vehicle to aripiprazole (*n* = 7)
LPS + chlorpromazine (*n* = 4)	LPS + quetiapine (*n* = 9)	LPS + aripiprazole (*n* = 7)

**Table 2:** The impact of prenatal exposure to LPS and subsequent chronic treatment with chlorpromazine (**A**), quetiapine (**B**) or aripiprazole (**C**) on *Cx3cl1*, *Cx3cr1*, *Cd200* or *Cd200r* gene expression in the frontal cortices of the offspring. The mRNA levels were measured using qRT-PCR with *n* = 4–6 (chlorpromazine), *n* = 5–9 (quetiapine) and *n* = 5–6 (aripiprazole) in each group. The results are presented as the average fold change ± SEM. * *p* < 0.05 vs. control + vehicle group.

**(A) Cohort 1—chlorpromazine**				
**Factor**	**Gene Expression in the Frontal Cortices**			
	**Control**		**LPS**	
	**vehicle**	**chlorpromazine**	**vehicle**	**chlorpromazine**
*Cx3cl1*	1.38 ± 0.57	1.75 ± 0.71	5.27 ± 1.20 *	7.16 ± 1.16
*Cx3cr1*	1.28 ± 0.46	1.46 ± 0.60	5.39 ± 1.83 *	8.29 ± 1.82
*Cd200*	1.28 ± 0.43	1.67 ± 0.71	4.34 ± 1.23 *	4.75 ± 1.11
*Cd200r*	1.33 ± 0.46	1.74 ± 0.50	13.24 ± 4.58 *	11.45 ± 3.03
**(B) Cohort 2—quetiapine**				
**Factor**	**Gene Expression in the Frontal Cortices**			
	**Control**		**LPS**	
	**vehicle**	**quetiapine**	**vehicle**	**quetiapine**
*Cx3cl1*	1.01 ± 0.06	0.94 ± 0.12	1.09 ± 0.10	1.12 ± 0.10
*Cx3cr1*	1.01 ± 0.04	1.27 ± 0.12	1.51 ± 0.23	1.48 ± 0.25
*Cd200*	1.05 ± 0.16	0.42 ± 0.12	1.33 ± 0.41	1.50 ± 0.32
*Cd200r*	1.12 ± 0.22	0.60 ± 0.24	2.95 ± 0.65 *	2.28 ± 0.71
**(C) Cohort 3—aripiprazole**				
**Factor**	**Gene Expression in the Frontal Cortices**			
	**Control**		**LPS**	
	**vehicle**	**aripiprazole**	**vehicle**	**aripiprazole**
*Cx3cl1*	1.05 ± 0.15	1.20 ± 0.21	1.58 ± 0.28	1.87 ± 0.26
*Cx3cr1*	1.04 ± 0.13	0.92 ± 0.12	1.00 ± 0.20	1.19 ± 0.16
*Cd200*	1.04 ± 0.12	1.16 ± 0.15	1.20 ± 0.17	1.29 ± 0.13
*Cd200r*	1.10 ± 0.22	1.79 ± 0.43	2.61 ± 0.53 *	2.14 ± 0.34

**Table 3:** The impact of prenatal exposure to LPS and subsequent chronic treatment with chlorpromazine (**A**), quetiapine (**B**) or aripiprazole (**C**) on *Cd40*, *Cd68*, *Arg1* and *Igf-1* gene expression in the frontal cortices of the offspring. The mRNA levels were measured using qRT-PCR with *n* = 4–6 (chlorpromazine), *n* = 6–9 (quetiapine) and *n* = 5–6 (aripiprazole) in each group. The results are presented as the average fold change ± SEM. * *p* < 0.05 vs. control + vehicle group, # *p* < 0.05 vs. LPS + vehicle group.

**(A) Cohort 1—chlorpromazine**				
**Factor**	**Gene Expression in the Frontal Cortices**			
	**control**		**LPS**	
	**vehicle**	**chlorpromazine**	**vehicle**	**chlorpromazine**
*Cd40*	1.41 ± 0.53	1.14 ± 0.45	1.41 ± 0.53	1.14 ± 0.45
*Cd68*	1.33 ± 0.37	1.19 ± 0.40	1.33 ± 0.37	1.19 ± 0.40
*Arg1*	1.77 ± 0.71	1.96 ± 0.76	1.77 ± 0.71	1.96 ± 0.76
*Igf-1*	1.12 ± 0.19	1.30 ± 0.25	1.12 ± 0.19	1.30 ± 0.25
**(B) Cohort 2—quetiapine**				
**Factor**	**Gene Expression in the Frontal Cortices**			
	**control**		**LPS**	
	**vehicle**	**quetiapine**	**vehicle**	**quetiapine**
*Cd40*	1.02 ± 0.06	0.86 ± 0.11	1.27 ± 0.14	1.02 ± 0.16
*Cd68*	0.87 ± 0.05	0.73 ± 0.09	1.18 ± 0.11 *	**0.86 ± 0.09 ^#^**
*Arg1*	1.12 ± 0.23	0.36 ± 0.11	2.04 ± 0.52	2.29 ± 0.47
*Igf-1*	1.00 ± 0.03	0.75 ± 0.09	1.15 ± 0.19	1.27 ± 0.22
**(C) Cohort 3—aripiprazole**				
**Factor**	**Gene Expression in the Frontal Cortices**			
	**control**		**LPS**	
	**vehicle**	**aripiprazole**	**vehicle**	**aripiprazole**
*Cd40*	1.01 ± 0.06	0.88 ± 0.11	1.30 ± 0.17	1.35 ± 0.24
*Cd68*	1.01 ± 0.07	0.96 ± 0.09	0.96 ± 0.16	1.10 ± 0.22
*Arg1*	1.06 ± 0.15	1.48 ± 0.22	1.39 ± 0.16	1.56 ± 0.17
*Igf-1*	1.01 ± 0.05	1.06 ± 0.02	1.27 ± 0.08 *	1.26 ± 0.07

**Table 4:** The impact of prenatal exposure to LPS and subsequent chronic treatment with quetiapine on the gene expression of *Cx3cl1*, *Cx3cr1*, *Cd200* and *Cd200r* (**A**) as well as *Cd40*, *Cd68*, *Arg1* and *Igf-1* (**B**) in the hippocampi of the offspring. The mRNA levels were measured using qRT-PCR with *n* = 7–8 in each group. The results are presented as the average fold change ± SEM.

(A)				
**Factor**	**Gene Expression in the Hippocampi**			
	**control**		**LPS**	
	**vehicle**	**quetiapine**	**vehicle**	**quetiapine**
*Cx3cl1*	1.02 ± 0.08	1.56 ± 0.27	0.97 ± 0.16	1.31 ± 0.25
*Cx3cr1*	1.10 ± 0.18	1.91 ± 0.37	1.17 ± 0.26	1.47 ± 0.28
*Cd200*	1.02 ± 0.07	1.12 ± 0.09	1.03 ± 0.11	1.13 ± 0.14
*Cd200r*	1.20 ± 0.29	1.90 ± 0.43	1.28 ± 0.34	1.64 ± 0.25
(**B**)				
**Factor**	**Gene Expression in the Hippocampi**			
	**control**		**LPS**	
	**vehicle**	**quetiapine**	**vehicle**	**quetiapine**
*Cd40*	1.05 ± 0.13	1.42 ± 0.23	1.07 ± 0.20	1.34 ± 0.24
*Cd68*	1.02 ± 0.07	1.26 ± 0.06	1.02 ± 0.14	1.20 ± 0.17
*Arg1*	1.04 ± 0.11	1.27 ± 0.14	1.11 ± 0.09	1.32 ± 0.11
*Igf-1*	1.02 ± 0.08	1.27 ± 0.12	1.04 ± 0.10	1.35 ± 0.19

## Data Availability

All data supporting the conclusions of this manuscript are provided in the text, figures and tables.
